# The Assessment of the Autonomic Polyneuropathy Through Sudoscan and Vitamin B12 in Patients with Type 2 Diabetes Mellitus and High Cardiovascular Risk or Established Cardiovascular Disease

**DOI:** 10.3390/biomedicines14010018

**Published:** 2025-12-21

**Authors:** Cristina Mocanu (Chitan), Teodor Salmen, Anca Pantea Stoian, Cristian Serafinceanu

**Affiliations:** 1 Doctoral School, Carol Davila University of Medicine and Pharmacy, 020021 Bucharest, Romania; cristina.mocanu-chitan@drd.umfcd.ro; 2 Outpatient Diabetes Center 1, Suceava County Emergency Clinical Hospital “Sf. Ioan cel Nou”, 720224 Suceava, Romania; 3 Department of Diabetes, Nutrition and Metabolic Diseases, “Carol Davila” University of Medicine and Pharmacy, 020021 Bucharest, Romania; anca.stoian@umfcd.ro (A.P.S.); cristian.serafinceanu@umfcd.ro (C.S.)

**Keywords:** diabetes mellitus, diabetic peripheral neuropathy, ortostatin hypotension, Sudoscan, Vitamin B12

## Abstract

**Background:** Diabetes Mellitus (DM) is frequently associated with diabetic peripheral neuropathy (DPN) and cardiovascular diseases (CVD). The aim of this study is to assess the relationship between DPN symptoms, vitamin B12 level, and autonomic neuropathy in DM patients with high and very high CV risk or established CVD. **Material and Methods:** A cross-sectional analysis of 164 patients from the Outpatient DM Department of Suceava County Hospital from September 2025 was performed. The clinical, paraclinical, and demographic data were collected, including Toronto Clinical Neuropathy Score (TCNS), Sudoscan, Orthostatic Hypotension (OH), and B12 level. **Results:** In total, 65.9% of patients had DPN; the mean HbA1c was 8.22% ± 1.74. No significant correlation was obtained between autonomic neuropathy (Sudoscan) and DPN severity (*p* = 0.163) or between vitamin B12 and DPN (*p* = 0.6). Vitamin B12 was associated with CV risk assessed with Sudosan (*p* = 0.04). OH had limited diagnostic significance for autonomic dysfunction. **Conclusions:** No strong link was detected between B12 levels and DPN; thus, it cannot be considered a predictive marker. Objective DPN screening remains essential. Sudoscan is practical and non-invasive in assessing autonomic neuropathy, but only when combined with TCNS may it increase the DPN screening and risk stratification in high-CV-risk populations with DM.

## 1. Introduction

Diabetes mellitus (DM) is a chronic condition, alongside cardiovascular diseases (CVD), cancers, and chronic pulmonary diseases, that, altogether, are the modern pandemic of chronic non-transmissible diseases [1]. DM has a prevalence that is reported by the 2025 International Diabetes Federation Atlas to grow significantly from 2024 until 2050, from 45% to over 850 million cases [2]. Moreover, the American Diabetes Association characterizes DM as a metabolic disorder that, in the case of poor glucose control, has a severe impact on health, reflected by chronic, long-term developed complications such as diabetic polyneuropathy (DPN), retinopathy, CVD, heart disease, peripheral artery disease, acute myocardial infarction, stroke, and chronic kidney disease, or by acute life-threatening events such as diabetic ketoacidosis [3,4,5].

DPN is a common and debilitating complication of DM, which places a high burden on healthcare systems worldwide due to complications such as ulcerations and limb amputations, which require large recovery periods and costs. An early diagnosis and prevention through optimal glycemic control are essential to reduce the burden of this condition. Sensory neuropathy often presents insidiously but can progress to severe neuropathic pain, which adversely affects quality of life by impairing mobility and increasing the risk of foot ulcers and amputations. Despite the known benefits of glycemic control and lifestyle interventions in delaying DPN onset, pharmacologic therapy is a main part of the management strategy for established neuropathic pain both in DM and prediabetes populations [6,7,8].

Landmark studies, including the Stockholm Diabetes Intervention Study and the Diabetes Control and Complications Trial, prove that tight glycemic control can effectively prevent or modestly slow the progression of DPN and cardiovascular (CV) autonomic neuropathy, but without reversing neuronal loss, a fact that cannot be achieved even with disease-modifying treatment [9].

The diagnosis of DPN remains challenging in the absence of a gold standard method, even though we have a variety of clinical and electrophysiological assessments and electrodiagnostic testing, which is less practical in routine clinical settings. Among screening tools, the Toronto Clinical Neuropathy Score (TCNS) is widely used, while autonomic testing often relies on CV reflex testing, such as orthostatic blood pressure (BP) measurements and emerging tools, including Sudoscan, a rapid, non-invasive assessment of both small fiber and autonomic function [10,11,12].

Patients classified in the high and very high CV risk category or with confirmed CVD usually present with severe atherosclerotic processes with complex systemic lesions, which include endothelial dysfunction, microvascular lesions, and neurological alterations. On the other hand, DPN is frequently associated not only with macrovascular complications but also with microvascular complications, but without a clearly understood pathophysiologic mechanism. The endothelial dysfunction in patients with high and very high CV risk or with CVD occurs early in DM and is linked to CV risk factors and DPN [13,14,15,16,17].

Because vitamin B12 deficiency has been cited in DPN development and progression and is often underdiagnosed and because there is a gap in stratification of patients with DM and CV risk, for a better evaluation of the global risk and a better optimization of therapeutic and prevention strategies, the aim of this study is to evaluate, in patients with DM and high and very high CR risk or with established CVD, the relationship between symptoms of DPN, as measured by the TCNS and autonomic neuropathy, evaluated by Sudoscan, orthostatic hypotension (OH) and serum vitamin B12 levels.

## 2. Materials and Methods

A cross-sectional evaluation of consecutively presented patients with DM and high and very high CV risk or established CVD from the Diabetes Outpatient Department of Suceava Emergency County Hospital, from two diabetologists, between 1 and 30 September 2025, with 5 outpatients’ days a week.

The study was reviewed according to the Helsinki Declaration and received ethical approval by the Ethical Committee of the Emergency County Clinical Hospital “Sfântul Ioan cel Nou” Suceava, number 36/28.08.2025. A total of 196 patients were included and evaluated to determine if they met the inclusion and exclusion criteria, which are summarized in Table 1.

CVD is represented by a history of acute coronary syndrome, such as acute myocardial infarction, stable or unstable angina pectoris, or a history of arterial revascularization of coronary arteries or of other nature, acute or transient stroke, or peripheral artery disease, including aortic aneurysm [18]. Also, a high or very high CV risk category was defined according to the SCORE2-Diabetes Tool [19,20,21].

Only 164 patients with high and very high CV risk or established CVD and DM that met the criteria were included and were evaluated by the same specialist in DM by demographic (gender, age, settlement), clinical (height and weight for body mass index (BMI), both clinostatic and orthostatic BP), medical history (DM characteristics such as duration and complications and comorbidities), and paraclinical (HbA1c, CV risk, Sudoscan [22], TCNS [23], vitamin B12 seric level) data. The paraclinical evaluation, made by the diabetologist, included à jeun blood sampling results that were evaluated in the last month, and further, the patients were evaluated on the same day but not at a specific time of the day, in the same consulting room, at a constant temperature of around 20–22 °C by TCNS and then by Sudoscan. TCNS evaluated limb symptoms by 0—normal and 1—abnormal or weak; feel by 0—normal and 1—abnormal or weak; and reflex by 0—normal, 1—abnormal or weak, or 2—disappear. TCNS scoring is from 0 to 19, respectively: 0–5 no DPN, 6–8 mild DPN, 9–11 moderate DPN, and 12–19 severe DPN.

Furthermore, the patients with DM were divided into two groups: the first group included patients who had symptoms of DPN with/without associated treatment and the second group of patients who did not declare symptoms of DPN. The diagnosis of DPN is made by clinical symptomatology, which is confirmed by a neurologist.

OH was evaluated according to its definition; a decrease in systolic BP by 20 mmHg or a decrease in diastolic BP by 10 mmHg maintained over 3 min after moving from lying down to standing. Vitamin B12 deficiency was defined as a value below 148 pmol/L [24].

Sudoscan evaluates neuropathy reflected by sudomotor dysfunction using electrochemical skin conductance (ESC), measured in microSiemens (µS), and is the ratio of generated current to a constant DC stimulus (≤4 V) applied to electrodes. The ESC values are categorized as >60 µS for no dysfunction, 60–40 µS for moderate dysfunction, and <40 µS for severe dysfunction [22].

The DM evaluation criteria of the included patients were undertaken according to the standards presented in the national guidelines, which are the same as those of the American Diabetes Association Standards of Care [25].

### Statistical Analysis

Statistical analyses were performed using IBM SPSS Statistics for Windows, version 20.0 (IBM, Armonk, NY, USA). The continuous variables were evaluated for normality of distributions, and the normally distributed variables were expressed as mean and standard deviation (SD), whereas non-normally distributed variables were presented as median and interquartile range. The categorical variables were reported as absolute frequencies and percentages. Statistical significance was assessed using a 95% confidence interval (CI). Analysis of variance, ANOVA, was used to compare quantitative variables between groups. All statistical tests were two-tailed, and an accepted *p*-value < 0.05 was considered statistically significant. For correlations, we used Pearson and Levene tests.

## 3. Results

### 3.1. Baseline Characteristics

The demographic, clinical, and DM characteristics and comorbidities of the 164 included patients are summarized in Table 2.

### 3.2. Neuropathy Findings

The correlation coefficient between the CV risk calculated by SCORE2-Ddiabetes and the CV risk estimated by Sudoscan is 0.098.

**Figure 1:**
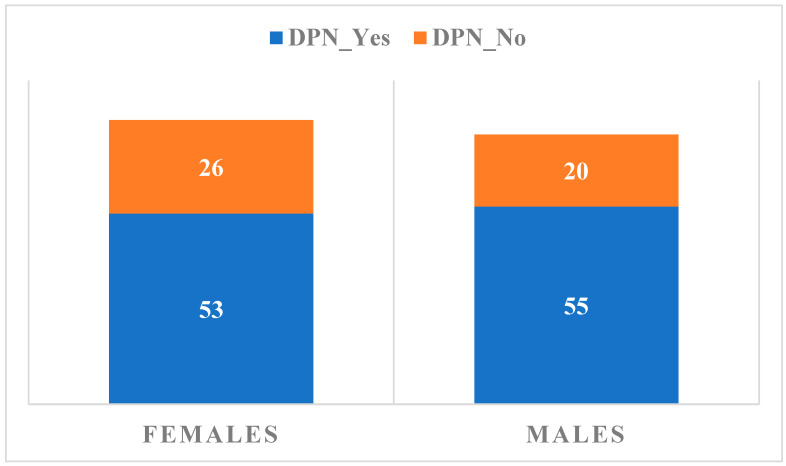
Distribution of subjects according to gender and the presence or absence of confirmed DPN.

The patients’ distribution according to gender and the presence/absence of confirmed DPN is summarized in Figure 1.

**Figure 2:**
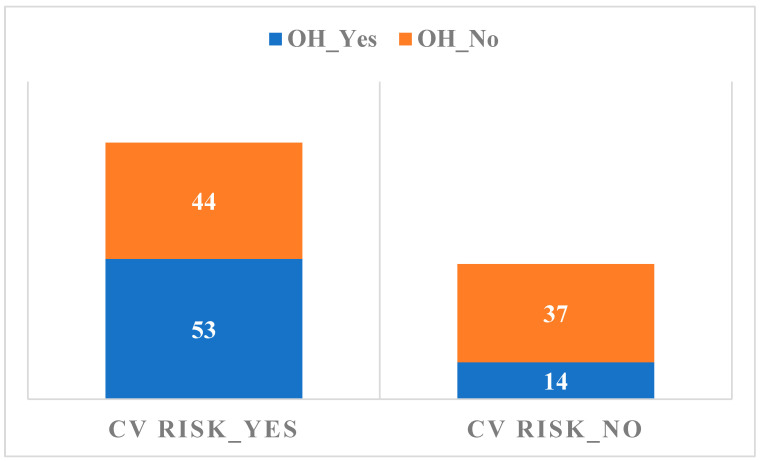
The relationship between the presence of OH and the CV risk.

The relationship between the presence of OH and the CV risk calculated by the Sudoscan medical device is summarized in Figure 2.

**Figure 3:**
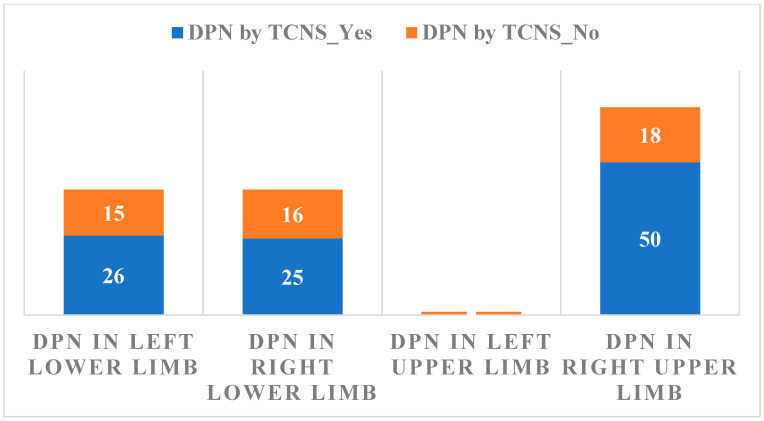
The relationship between DPN evaluated by TCNS results and Sudoscan measurements.

The relationship between DPN evaluated by TCNS and the changes in Sudoscan measurements are summarized in Figure 3.

Considering Sudoscan as a measure of autonomic neuropathy, its association with DPN evaluated by TCNS results is not statistically significant, *p* = 0.163.

### 3.3. Correlations

**Figure 4:**
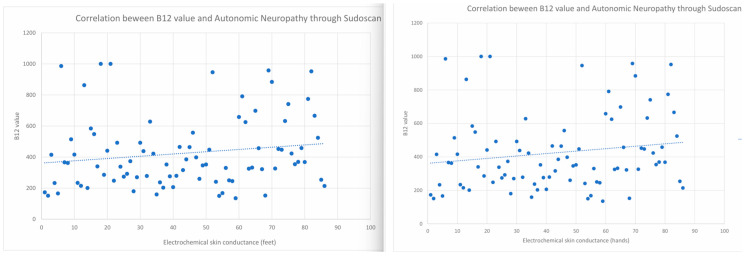
The relationship between levels of Vitamin B12 and the presence of autonomic neuropathy was evaluated by Sudoscan (left for feet and right for hands).

A high degree of variability in vitamin B12 levels was observed across both groups, with values ranging from 0 to 1000 pg/mL. Statistical analysis of its association with autonomic neuropathy evaluated by Sudoscan yielded a *p*-value of 0.6, a result of no statistical significance, as illustrated in Figure 4.

The correlations between the presence of autonomic neuropathy as evaluated by Sudoscan and Vitamin B12 are as follows—r = 0.038, 95%CI (−0.122, 0.196) for right lower limb, r = 0.002, 95%CI (−0.158, 0.161) for left lower limb, r = −0.061, 95%CI (−0.218, 0.099) for left upper limb and r = −0.028, 95%CI (−0.186, 0.132) for right upper limb. The association between DM duration and autonomic neuropathy, as evaluated by Sudoscan, was 1.07189, 95% CI (0.807, 1.211) in the Levene test.

## 4. Discussion

Evaluation of patients with high or very high CV risk or established CVD for the presence of DPN allows a more precise stratification of the overall risk and an optimization of therapeutic and prevention strategies. Thus, we proceed to analyze, in patients with DM and high and very high CV risk or established CVD, the relationship between symptoms of peripheral DPN—measured by the TCNS and autonomic neuropathy—evaluated by Sudoscan, OH, and serum vitamin B12 levels.

CVD is the main cause of morbidity and mortality in patients with DM, while DM is an independent risk factor for CVD. Also, in patients with DM, all main CV risk factors, such as high BP, dyslipidemia, or obesity, are common [18,26]. The presence of confirmed CVD without regard to the treatment was observed in 61% of the included patients, while the other 39% were at high and very high CV risk according to the SCORE2-Diabetes calculation formula. The correlation between the CV risk calculated by SCORE2 Diabetes expresses a low degree of association but similar positive incremental trends [20].

The age criterion, specifically the maximum limit of 80 years, is due to the increased prevalence of non-DM causes of polyneuropathy such as nutritional deficiencies, drug-induced B12 deficits, or multiple associated comorbidities, while the maximum evolution of DM of 10 years is due to the fact that after this point the DPN is most probably established and the diagnostic utility of the tests decreases. According to the data in Table 1, females are, on average, older than males, with a greater variation in age for males compared to females. Mean BMI is slightly higher in females than in males, indicating a slightly greater trend toward overweight/obesity in females in this sample, with a greater variability in BMI for females compared to males. Insulin therapy was initiated from the DM onset in 24% of the included patients; further, at the time of evaluation, 38.6% of patients were treated with insulin.

Thus, we managed to provide a better view of the DPN and to limit other possible etiologic factors because our population is equally distributed as gender and settlement and is one of great interest, due to a relatively young age (mean age of 63 years), with a relatively short mean DM duration (7 years), with CV risk factors such as obesity (mean BMI of 31 kg/m^2^), HBP (mean SBP of 140 mm Hg) and relatively controlled DM (mean HbA1c = 8.22%). Moreover, this is a period when complications begin to develop and when screening and diagnostic tests are very important.

There is no single specific measurement to reliably diagnose or rule out the presence of vitamin B12 deficiency. A serum vitamin B12 concentration below 148 pmol/L, in combination with symptoms, is a strong indication of deficiency and is sufficient to recommend initiating supplementation treatment [27,28,29]. However, symptoms may also be present in individuals with serum vitamin B12 values >148 pmol/L. Vitamin B12 supplementation can lead to serum concentrations in the “normal” range or sometimes above the “normal” level without reducing symptoms, which masks the correct diagnosis [27,28,29].

A position statement of the American Diabetes Association recommends an annual 10 g monofilament test screening in patients with type 2 DM, for predicting foot ulceration and amputation, but without sensitivity for the early detection of DPN [6,7,12,27]. Analyzing symptomatic DPN and taking into consideration gender distribution, we observed 56 patients (26 females and 30 males), representing 34% of the total, without specific signs of DPN, as compared to 108 patients (53 females and 55 males), representing 66% of the total, with signs present or symptomatic status in the last 3 months.

Our analysis found that the 10 g monofilament test plus Sudoscan was better for detecting DPN, but the Sudoscan evaluation plus determination of vitamin B12 or OH did not improve the diagnostic power compared with the TCNS. Sudoscan can measure foot ESC within 3 min and needs no special preparation. Recent studies have demonstrated that the Sudoscan assessment is a sensitive tool for detecting DPN in patients with DM, with a sensitivity of 78% and specificity of 92%, a performance that is clinically equivalent to or better than traditional DPN scores [10,11,30].

Autonomic polyneuropathy is a microvascular complication resulting from damage to the sympathetic and parasympathetic nerve fibers that innervate the heart muscle and blood vessels, favoring changes in CV autonomic control. The mechanism of tension collapse is secondary to the effect of gravity, respectively, the need to translocate 500 to 700 mL of blood from the upper part of the body to the vessels of high venous capacity of the lower limbs and the splanchnic circulation, rapidly, in a few seconds, under conditions of altered vasoconstriction function; with the main causes being represented by the failure of noradrenergic neurotransmission. Neurogenic OH in patients with DM is usually accompanied by secondary autonomic dysregulation and may also involve other organ systems, such as the intestine and bladder, causing gastroparesis and erectile dysfunction as well as changes in sweating regulation [31,32,33].

OH can be modified by other associated CV pathologies or by factors such as age, polypharmacy, hypovolemia, smoking status, high BP (treated or not), the presence of hydrostatic varicose veins, or other neurological diseases. The prevalence of autonomic polyneuropathy is reported of 20% among patients with DM and, respectively, 30% among patients with Parkinson’s disease or dementia [34]. OH is a microvascular complication that is an important risk factor for silent myocardial ischemia, chronic kidney disease, myocardial dysfunction, major CV events, cardiac arrhythmias, and sudden death, and also, is a late sign that the disease of the sympathetic nervous system may be secondary to causes other than neurological ones [35,36,37].

The relationship between the presence of OH and the CV risk evaluated by Sudoscan is not significant, considering that more patients, both from the OH and without OH groups, present with an increased CV risk following Sudoscan recordings. There is not enough evidence to support the idea that there is a significant difference between groups or conditions on the autonomic neuropathy factor evaluated by Sudoscan as compared to TCNS results, *p* = 0.163. Sudoscan, from the recent studies and from the evaluations carried out in this paper, has a significant statistical significance compared to the TCNS/monofilament testing for the early diagnosis of peripheral DPN, as well as autonomic neuropathy (*p* = 0.001) [22,30].

A significant correlation was observed between the TCNS and the Sudoscan conductance values (Figure 3), suggesting that higher DPN symptom scores are associated with impaired sudomotor function. The presence of DPN is associated with lower values of Sudoscan determination (95% CI ranges from 65 to 75) compared to its absence of DPN (95% CI ranges from 75 to 85), indicating a statistically significant difference between the two groups. The analysis of the relationship between CV risk (Sudoscan determination) and the presence of autonomic polyneuropathy (the presence of OH) reveals no significant difference in CV risk between those with and without autonomic polyneuropathy. The wide distribution and overlapping mean values of CV risk in both groups indicate that other factors may play a more crucial role in determining CV risk than the presence of autonomic polyneuropathy alone. Based on the *p*-value (0.040), there is sufficient evidence to conclude that there are significant differences between the groups in terms of CV risk. The F value (1.63) supports this conclusion, indicating that between-group variation is greater than within-group variation. Thus, we can say that the compared groups have a significant effect on CV risk in the analyzed data. This result is important for understanding the factors that influence CV risk and may guide clinical interventions or prevention strategies. OH is a parameter that cannot be used as a single determination, having a low statistical significance in the diagnosis of autonomic neuropathy (*p* = 0.163)

The dosage of vitamin B12 cannot be a diagnostic predictive marker, demonstrating a reduced statistical significance in relation to the TCNS/Monofilament testing (*p* = 0.700/0.610), but also with autonomic neuropathy tested by determining OH (*p* = 0.151). On the other hand, vitamin B12 and CV risk calculated by Sudoscan have an association *p* = 0.040. Vitamin B12 Mean: For the group without monofilament-confirmed DPN, the mean appears to be around 400. For the group with confirmed monofilament DPN, the mean also appears to be around 400 [37]. These findings suggest that there is no significant association between vitamin B12 levels and the presence of DPN as assessed by monofilament testing. Based on this analysis, the presence or absence of DPN does not appear to exert a significant effect on vitamin B12 concentrations. Based on descriptive statistics and CIs, there is evidence that mean scores differ significantly between groups with and without DPN, confirmed by the monofilament sensitivity test. The lower mean in the DPN group suggests that these patients may have a lower level of tactile sensitivity, indicating the possibility of DPN [38,39,40].

Determinations obtained with Sudoscan at the level of the feet and hands are highly correlated with each other, especially the measurements of the feet with each other and those of the hands with each other. However, there is no significant correlation between these determinations and the level of Vitamin B12, indicating that the level of Vitamin B12 does not directly influence the presence or absence of Sudoscan-supported neuropathy.

Recently, a meta-analysis did not find enough evidence to associate DPN and vitamin B12 deficiency, but there are not enough quality studies to obtain conclusive results. Another study in which vitamin B12 supplementation was performed in patients with diabetic PDN found no conclusive evidence regarding improvement of DPN symptoms [26]. Interestingly, folic acid supplementation does appear to have a positive effect on DPN according to a recent study [6]. 

Low serum levels of vitamin B12 are strong predictors and risk factors for the presence and severity of DPN in patients with DM, especially with high or very high CV risk or established CVD and with worse DPN symptoms. Neuropathy from B12 vitamin deficiency mimics or worsens DPN symptoms. Metformin use, very frequent in patients with DM, can lead to a dose- and duration-dependent deficiency. Also, older age and longer duration of DM are strong independent predictors of vitamin B12 deficiency in DPN patients. On the other hand, high levels of B12 vitamin are associated with increased CVD mortality risk [27,41,42,43,44].

The clinical implication, as the American Diabetes Association and other clinical guidelines recommend, is to establish an annual monitoring of vitamin B12 levels for all patients on chronic metformin therapy, especially those with existing neuropathy or other risk factors. Identifying and treating B12 deficiency is crucial because B12 deficiency-induced neuropathy can be prevented or even reversed with supplementation, which is not always the case for DPN caused solely by DM. Vitamin B12 supplementation has been shown to improve neurophysiological parameters, reduce pain scores, and enhance quality of life in DPN patients with low or borderline B12 levels [27,41,42].

Also, Sudoscan can screen DPN and cardiac autonomic neuropathy quickly and in an objective manner, with limitations such as the moderate specificity of Sudoscan for cardiac autonomic neuropathy, so it requires adjuvant methods [8,45].

Despite the extensive literature on DPN, there is a lack of integrated assessment of somatic and autonomic neuropathy and vitamin B12 levels specifically in patients with type 2 DM and high or very high CV risk or established CVD. Most prior studies have evaluated these components independently, in heterogeneous or lower-risk populations, and rarely in a combined, real-world outpatient setting focused on advanced CV risk. Thus, there is limited evidence on how autonomic dysfunction detected by emerging tools such as Sudoscan interacts with clinical DPN severity and vitamin B12 levels in this particularly vulnerable subgroup. Our study directly addresses this gap by providing a simultaneous evaluation of TCNS-defined DPN, Sudoscan-derived autonomic dysfunction OH, and vitamin B12 status in a high-CV-risk population with DM.

With respect to current screening strategies, Sudoscan contributes objective, rapid, and non-invasive identification of small-fiber and autonomic dysfunction, which is not captured by conventional tools such as the 10 g monofilament or symptom-based scores alone. While traditional methods primarily detect advanced large-fiber neuropathy, Sudoscan enables earlier detection of sudomotor dysfunction, a surrogate for early autonomic and small-fiber involvement. Our findings demonstrate that Sudoscan adds incremental value when combined with TCNS, improving functional risk stratification of neuropathy in patients already burdened by high CV risk, whereas orthostatic hypotension alone showed limited diagnostic utility. Beyond current American Diabetes Association recommendations, which recommend annual monofilament testing primarily for ulcer and amputation risk, our findings support a more refined, multimodal DPM screening approach in high-risk CV patients with DM. The lack of a strong association between vitamin B12 levels and either somatic or autonomic neuropathy underscores that routine B12 measurement alone is insufficient for DPN risk stratification. Therefore, the use of TCNS and Sudoscan allows clinicians to identify patients with early autonomic involvement who may otherwise remain undetected using standard recommended screening alone. This has direct implications for earlier preventive interventions, intensification of glycemic and CV risk control, and closer monitoring for silent autonomic complications, thereby advancing clinical decision-making toward a more personalized, risk-adapted approach in complex DM patients with elevated CV risk.

### 4.1. Further Directions

Further research could investigate additional variables that may influence CV risk, such as lifestyle factors, genetic predispositions, and other comorbidities, and also additional factors that may influence vitamin B12 levels, such as dietary intake, overall health status, and results from other relevant medical or biochemical assessments. Prospective studies are needed to evaluate whether this newly developed scoring system using Sudoscan can predict foot ulceration or amputation. We will evaluate the novel electrocardiographic parameter designed to improve heart rate variability assessment in aging populations, that in patients with DM, refers to the T–R interval [46].

### 4.2. Limitations

The limitations of our study are the exclusion of patients with chronic diseases such as amyloidosis, Parkinson’s disease with or without treatment, and hemiparesis/hemiplegia after stroke. The diagnosis of DPN was performed using heterogeneous methods, including clinical history, clinical or electrophysiological evaluation, or the TCNS. These methods vary in sensitivity and specificity, which can affect the reliability of the diagnosis. Furthermore, some tests assessing neuropathy risk factors and sensitive nerve function are subjective and may be influenced or misinterpreted by either the patient or the evaluator. The methodology of this study did not allow us to establish a causal association between vitamin B12 deficiency and diabetic DPN. Additionally, the analysis did not account for the lack of cofounders of DPN evaluation—age, DM duration, comorbidities, CV risk, or insulin use, and the correlation was unadjusted.

## 5. Conclusions

Sudoscan evaluation at the feet and hands is highly correlated with each other, especially the measurements of the feet with each other and those of the hands with each other. However, there is no significant association between these determinations and the level of Vitamin B12, indicating that the level of Vitamin B12 does not directly influence the presence or absence of Sudoscan-supported DPN. OH has low statistical significance in the diagnosis of autonomic neuropathy. Our findings suggest that the 10 g monofilament test plus Sudoscan may be practical and accurate for detecting DPN in real clinical settings in patients with high or very high CV risk or established CVD.

Vitamin B12 levels in patients with high or very high CV risk or established CVD are similar between the groups with and without autonomic neuropathy, so the dosage of vitamin B12 cannot be a diagnostic predictive marker demonstrating a reduced statistical significance in relation to the TCNS/Monofilament testing and with autonomic neuropathy as assessed by OH, even when considered alongside the CV risk calculated by Sudoscan.

## Figures and Tables

**Table 1:** Inclusion and exclusion criteria.

Inclusion Criteria	Exclusion Criteria
Adults over 18 years old	Adults over 80 years old
Diagnosis of DM for at least 1 year	Diagnosis of DM for more than 10 years
High and very high cardiovascular risk or established cardiovascular disease	Moderate, low, or absent cardiovascular risk
Complete biologic profile	History of chronic diseases such as amyloidosis, Parkinson’s disease with or without treatment, hemiparesis/hemiplegia after stroke diseases, lower limb amputation, or presence of plantar lesions/ulcerations
Informed consent signed	

DM—Diabetes Mellitus.

**Table 2:** Demographic, clinical, and DM characteristics and comorbidities of the included patients.

Characteristic	*n* = 164	95% CI
Demographic characteristics		
Male gender, *n*, (%)	79, (48.17%)	
Age (years), mean ± SD	63 ± 12.29	61.1, 64.9
Urban settlement, *n*, (%)	103, (62.81%)	
Clinical characteristics		
BMI (kg/m^2^), mean ± SD	31.37 ± 5.97	30.45, 32.29
Clinostatic BP (mmHg), mean ± SD	Systolic BP 143 ± 17.3	140.33, 145.67
	Diastolic BP 76.40 ± 11.39	74.64, 78.16
Orthostatic BP (mmHg), mean ± SD	Systolic BP 138 ± 17.8	135.26, 170.74
	Diastolic BP 72.780 ± 12.27	70.89, 74.67
DM characteristics		
DM type 2, *n*, (%)	156, (95.12%)	
DM duration, mean ± SD	7.22 ± 3.25	6.72, 7.72
Insulin-therapy, *n*, (%)	39, (23.78%)	
HbA1c (%), mean ± SD	8.22 ± 1.74	7.95, 8.49
DM complications		
High and Very High Cardiovascular Risk, *n*, (%)	66, (40.24%)	
Cardiovascular Disease, *n*, (%)	98, (59.75%)	
Diabetic Polyneuropathy, *n*, (%)	108, (65.85%)	
Neuropathic deformities/amputation of the limbs, *n*, (%)	37, (22.56%)	
Comorbidities		
Toxic/Viral hepatic ailments, *n*, (%)	11, (6.70%)	
Neoplastic ailments with chemotherapy, *n*, (%)	21, (12.80%)	
Neoplastic ailments with chemotherapy/radiotherapy, *n*, (%)	9, (5.48%)	
Thyroiditis (hypo/hyperthyroidism), *n*, (%)	19, (11.58%)	
Discopathy or other chronic rheumatological diseases, *n*, (%)	46, (28.04%)	

CI—confidence interval; SD—standard deviation; BMI—body mass index; BP—blood pressure; DM—Diabetes Mellitus.

## Data Availability

The raw data supporting the conclusions of this article will be made available by the authors on request.

## Abbreviations

The following abbreviations are used in this manuscript:

BMI	Body Mass Index
BP	Blood Pressure
CI	Confidence Interval
CV	Cardiovascular
CVD	Cardiovascular Disease
DM	Diabetes Mellitus
DPN	Diabetic Polyneuropathy
ESC	Electrochemical Skin Conductance
OH	Orthostatic Hypotension
SD	Standard Deviation
TCNS	Toronto Clinical Neuropathy Score
